# Perspectives of Patients With Dermatofibrosarcoma Protuberans on Diagnostic Delays, Surgical Outcomes, and Nonprotuberance

**DOI:** 10.1001/jamanetworkopen.2019.10413

**Published:** 2019-08-30

**Authors:** Marjorie Parker David, Ashley Funderburg, James P. Selig, Rebecca Brown, Pip M. Caliskan, Lee Cove, Gayle Dicker, Lori Hoffman, Tammi Horne, Jerad M. Gardner

**Affiliations:** 1 Department of Pathology and Laboratory Medicine, University of Texas Health Science Center at San Antonio; 2 Department of Pathology, University of Arkansas for Medical Sciences, Little Rock; 3 College of Nursing, University of Arkansas for Medical Sciences, Little Rock; 4 College of Public Health, University of Arkansas for Medical Sciences, Little Rock; 5 Dermatofibrosarcoma Protuberans Patient Advisory Board, International Group, College of Nursing, University of Arkansas for Medical Sciences, Little Rock; 6 Public Policy and External Affairs, Sarcoma Foundation of America, Damascus, Maryland

## Abstract

**Importance:**

Dermatofibrosarcoma protuberans (DFSP) may have a deceptively benign clinical appearance, including a nonprotuberant presentation. Patients with DFSP often perceive misdiagnoses and delays in receiving a diagnosis. Use of existing, patient-designed Facebook patient support groups (FBSGs) to recruit large numbers of patients with rare diseases may be an effective novel research method.

**Objectives:**

To collaborate with patients with rare disease through social media and answer questions important to both patients and the medical field, including sources of diagnostic delay, risk of recurrence, and flat presentation of DFSP.

**Design, Setting, and Participants:**

A multiple-choice survey created by a team of medical practitioners and patients with DFSP was administered to 214 patients with DFSP or family members from international DFSP FBSGs and a nonprofit foundation patient database via Lime Survey from October 30 to November 20, 2015. The survey asked questions designed to determine risk of recurrence and metastasis, surgical outcomes, sources of diagnostic delay, symptoms of recurrence, number of recurrences, scar size, and number of clinicians seen before biopsy. Statistical analysis was performed from January 1, 2016, to April 1, 2019.

**Main Outcomes and Measures:**

The study goal was to collect at least 200 survey responses.

**Results:**

Of 214 survey respondents (169 females and 45 males; mean [SD] age, 40.7 [12.1] years; range, <1 to 72 years), 199 were patients with DFSP and 15 were family members. Delays occurred between the patient noticing the DFSP lesion and receiving a diagnosis of DFSP (median, 4 years; range, <1 to 42 years). Most patients (112 [52.3%]) believed that they received a misdiagnosis at some point: by dermatologists (35 of 107 [32.7%]), primary care clinicians (80 of 107 [74.8%]), or another type of physician (27 of 107 [25.2%]). The most frequent prebiopsy clinical suspicion included cyst (101 [47.2%]), lipoma (30 [14.0%]), and scar (17 [7.9%]). Many patients first noticed their DFSP as a flat plaque (87 of 194 [44.8%]). Of these lesions, 73.6% (64 of 87) became protuberant eventually. Surgical treatments included Mohs micrographic surgery (56 of 194 [28.9%]), wide local excision (122 of 194 [62.9%]), and conservative excision (16 of 194 [8.2%]). The reported rate of recurrence was 5.4% (3 of 56) for Mohs micrographic surgery, 7.4% (9 of 122) for wide local excision, and 37.5% (6 of 16) for conservative excision. The higher rate of recurrence for conservative excision was significant (*P* = .001); there was no significant difference in the rate of recurrence between Mohs micrographic surgery and wide local excision (*P* = .76).

**Conclusions and Relevance:**

This study reports what appears to be disease-relevant statistics from the largest survey of patients with DFSP to date. Because of the dissonance between the name of the neoplasm and its clinical presentation, the alternative term *dermatofibrosarcoma, often protuberant* is proposed. This study suggests that FBSGs are useful tools in medical research, providing rapid access to large numbers of patients with rare diseases and enabling synergistic collaborations between patients and medical researchers.

## Key Points

**Question:**

Can dermatofibrosarcoma protuberans Facebook patient support groups work with researchers to determine risk of recurrence and metastasis, surgical outcomes, symptoms of recurrence, and sources of diagnostic delay?

**Findings:**

In this multiple-choice survey study, 214 patients with dermatofibrosarcoma protuberans reported a median of 4 years between noticing a lesion and receiving a correct diagnosis (range, <1 to 42 years), and many lesions (87 of 194 [44.8%]) first presented as flat. The mean age at noticing dermatofibrosarcoma protuberans was 29.6 years.

**Meaning:**

Facebook patient support groups seems to provide rapid access to large numbers of patients with rare diseases and enable synergistic collaborations between savvy patients and medical researchers.

## Teaser

This survey study collaborates with patients with dermatofibrosarcoma protuberans through social media to evaluate sources of diagnostic delay, risk of recurrence, and flat presentation of dermatofibrosarcoma protuberans.

## Introduction

Disease-specific Facebook support groups (FBSGs) are social media websites where members with rare diseases such as dermatofibrosarcoma protuberans (DFSP) can find many others with the same disease, whereas traditional in-person local support groups might have only a handful of members. One of us (J.M.G.) often discusses DFSP online with these groups.^1^ The idea for this study emerged from the suggestion of a DFSP FBSG member.^1^ This study also includes international respondents, allowing us to analyze global treatment approaches with a larger number of respondents.

Dermatofibrosarcoma protuberans is a rare sarcoma usually arising in or near the skin. It is often locally aggressive, sometimes recurring multiple times, but infrequently metastasizing. Dermatofibrosarcoma protuberans affects women at a slightly higher rate than men (53.1% in a study of 6817 individuals) and is diagnosed predominantly in individuals aged 20 to 59 years.^2^ Diagnostic delays are common, with a median delay of 3 to 5 years.^3,4^ At diagnosis, the mean lesion size is 4.4 to 4.9 cm^3,5^ and can require a large excision with a mean scar area of 21.7 cm^2^ with Mohs micrographic surgery to 63.4 cm^2^ with wide local excision (WLE).^6^ Nonprotuberance has been described among individuals with DFSP: 43% of patients first notice the lesion as nonprotuberant, and these patients experience a much longer interval to diagnosis (mean interval, 12.5 vs 8.1 years [n = 143]).^7^ The potential for the tumor name to cause misdiagnosis of flat lesions led us to propose an alternative terminology: *dermatofibrosarcoma, often protuberant* (DFSoP).

A subset of DFSoP tumors undergo higher-grade transformation into fibrosarcomatous DFSoP (FS-DFSoP). There is a substantially higher risk of recurrence in FS-DFSoP vs conventional DFSoP (29.8% vs 13.7%), as well as distant metastases (14.4% vs 1.1%).^8^

The National Comprehensive Cancer Network has recommended that surgical options for the treatment of DFSoP include Mohs micrographic surgery, WLE, and complete circumferential and peripheral deep margin assessment.^9^ With WLE, the local recurrence rate is reported to be from 0% to 30.8%,^3,5,6,10^ while Mohs micrographic surgery has a reported lower recurrence rate of 0% to 3.0%.^5,6,10,11^ However, Mohs micrographic surgery is not always available to patients. Complete circumferential and peripheral deep margin assessment, colloquially referred to as *slow Mohs*, is somewhat of a hybrid between WLE and Mohs micrographic surgery. In complete circumferential and peripheral deep margin assessment, the entire deep and circumferential margins are submitted for pathologic evaluation after surgery. The surgical site is kept open until the pathologic evaluation results are available.

Conservative excision (CE) tends to have the purpose of removing as much tumor as possible, particularly in a sensitive area such as the face or groin where the larger margins for a WLE or Mohs micrographic surgery may not be possible. Recurrences should be treated with additional surgery with the goal of negative surgical margins, and when this is not feasible, radiotherapy and/or imatinib therapy may be used.^9,12,13^

This large international survey study describes the diagnostic delays intrinsic to DFSoP and the clinical results of delay, the frequency of nonprotuberant presentation, the typical clinical presentation of DFSoP, and surgical outcomes. We also illustrate a novel research method using FBSGs to recruit large numbers of patients with rare diseases with existing patient-designed FBSGs and a partnership with the Sarcoma Foundation of America.

## Methods

A research collaboration was formed with 5 patient members of the DFSP FBSG (patient advisory board [PAB]), a representative from a sarcoma nonprofit organization, pathologists, a nurse scientist interested in community-based participatory research partnership, and research assistants experienced in medical anthropology and clinical research. The partnership completed online modules on ethical study design, data analysis, survey design, and communicating study results. Group members had regular videoconferences to discuss learning modules, survey design, survey questions, and survey administration. Multiple-choice questions of interest to patients and pathologists were designed based on patient posts in the DFSP FBSG and PAB member concerns and experiences, as well as traditional methods such as pathologists’ experience and literature review. This study was approved as exempt by the University of Arkansas for Medical Sciences Institutional Review Board because participation in the online survey was voluntary and the survey response data were deidentified and anonymous. As this was a voluntary online survey, a separate consent form was not required; participation in the survey represented consent to participate in the study. Respondents were informed at the beginning of the survey that their identification would remain anonymous to the researchers.

The survey was tested twice by each PAB member. The PAB members advertised the survey on a near-daily basis for 3 weeks by posting the survey advertisement on the 3 DFSP FBSGs with a goal of collecting 200 responses. Survey reminders were also sent privately to many DFSP FBSG members via Facebook Messenger. In addition, the nonprofit organization emailed advertisements to patients in their DFSP database. Study inclusion criteria were patients with DFSP or adults caring for someone with DFSP (including their child younger than 18 years), internet access, the ability to read and understand English, and age 18 years or older. Individuals excluded were those without internet access, those who could not read or understand English, and those younger than 18 years. Family members could respond to the survey on behalf of a relative, for example, if their child was younger than 18 years.

The American Association for Public Opinion Research (AAPOR) Standard Definitions for Surveys^14^ gives guidance for internet surveys of specifically named persons. There were no means to quantify how many patients viewed the survey advertisements on FBSGs, and survey responses were completely anonymous under the provisions of our exempt institutional review board approval status. Because of this, we are unable to calculate the response rate. Because of the anonymous survey and freely viewable advertisement, the AAPOR guidelines for internet surveys of specifically named persons does not apply to this study.

The survey was administered on the University of Arkansas for Medical Science’s servers using the survey platform Lime Survey. Before starting the survey, respondents were advised that they would need a copy of their pathology report, if available, and a ruler to measure scar size. There were a varied number of questions per respondent: respondents with metastases, recurrences, or other symptoms and experiences were prompted to answer additional questions pertinent to those scenarios: not all respondents answered all questions. The survey was open for 3 weeks, from October 30 to November 20, 2015. The survey instrument is available in the eAppendix in the Supplement.

### Statistical Analysis

Survey data were statistically analyzed from January 1, 2016, to April 1, 2019, using IBM SPSS Statistics software, version 25 (IBM Corp), and Microsoft Excel for Office 365 (Microsoft Corp). All statistical tests were 2-sided; we studied significant associations using Fisher exact test with *P* < .05 considered significant. To the best of our knowledge, this article conforms to the relevant aspects of the Strengthening the Reporting of Observational Studies in Epidemiology (STROBE) reporting guideline.

## Results

Seventeen partial surveys were submitted, 4 of which were excluded because they were found to have identical answers to a completed survey and were regarded as duplicates. Survey questions asked for the calendar year that events occurred, such as the year a lesion was first noticed by the patient and year of diagnosis. Time between these events was calculated by subtracting years. A few responses were inconsistent and were excluded (eg, respondent reporting the time of diagnosis occurring before going to the health care professional, resulting in a calculation of negative time between tumor onset and diagnosis).

### Participant Demographics

A total of 214 DFSP FBSG members and individuals in the nonprofit organization database (199 patients and 15 family members) worldwide responded to the survey, making this the largest survey of patients with DFSoP, to our knowledge. Respondents received treatment in the United States (117 of 201 [58.2%]), United Kingdom (32 of 201 [15.9%]), Canada (14 of 201 [7.0%]), Australia (13 of 201 [6.5%]), India (3 of 201 [1.5%]), Italy (3 of 201 [1.5%]), Germany (2 of 201 [1.0%]), and other countries (17 of 201 [8.5%]). Results were obtained from survey responses; no medical records or pathologic materials were reviewed. Participants ranged in age from younger than 1 year to 72 years at the time of survey completion (mean [SD] age, 40.7 [12.1] years); 169 participants (79.0%) were female.

### Diagnostic Delays, Misdiagnoses, and Prebiopsy Clinical Suspicion

The term *clinician* refers to medical professionals such as physicians, general practitioners, nurse practitioners, and physician assistants. Five respondents (2.3%) had lesions first detected by their clinician; 2 (0.9%) by imaging; 40 (18.7%) by a family member, partner, friend, or coworker; and most respondents (167 [78.0%]) detected their lesions themselves.

Respondents reported calendar year of their birth, year of noticing lesion, year of seeking care, and year of correct diagnosis. The mean (SD) age was 29.6 (12.2) years at first noticing a lesion, 33.0 (11.5) years at seeking care, and 36.7 (12.0) years (n = 213) at receiving a diagnosis of DFSoP.

The median time from the patient first noticing the tumor to visiting a clinician for evaluation was 1 year (range, <1 to 31 years [n = 209]). The median time from initial medical visit to receiving a diagnosis of DFSoP was less than 1 year (range, <1 to 41 years [n = 213]). The median total time from the patient first noticing the tumor to receiving the diagnosis of DFSoP was 4 years (range, <1 to 42 years [n = 212]).

Most patients (112 [52.3%]) believed they were misdiagnosed at some point, while 80 (37.4%) thought that they were never misdiagnosed and 22 (10.3%) were unsure. Of those who believed they were misdiagnosed, nearly all thought they were misdiagnosed before biopsy (107 of 112 [95.5%]; clinical misdiagnosis), while 17 of 112 (15.2%) thought they were misdiagnosed after biopsy (pathologic misdiagnosis), which includes 12 of 112 (10.7%) who thought they had a misdiagnosis both before and after biopsy (clinical and pathologic misdiagnosis). The potential clinical misdiagnoses were rendered by dermatologists (35 of 107 [32.7%]), primary care clinicians (80 of 107 [74.8%]), and other physicians (27 of 107 [25.2%]). Misdiagnoses could be reported from more than 1 type of clinician.

The most common prebiopsy clinical suspicions were cyst (101 [47.2%]), lipoma (30 [14.0%]), scar (17 [7.9%]), dermatofibroma or benign fibrous histiocytoma (13 [6.1%]), and keloid (8 [3.7%], among others (Table). Various forms of sarcoma were suspected in only 4 patients (1.9%). More than 1 prebiopsy suspicion could be reported because many individuals had multiple clinicians with different suspicions.

**Table.** Prebiopsy Clinical Experience for Patients With Dermatofibrosarcoma, Often Protuberant and Fibrosarcomatous Dermatofibrosarcoma, Often Protuberant^a^

Parameters	No. (%)
Prebiopsy clinical suspicion (n = 214)	
Cyst	101 (47.2)
Lipoma	30 (14.0)
Scar	17 (7.9)
Dermatofibroma or benign fibrous histiocytoma	13 (6.1)
Keloid	8 (3.7)
Birthmark	6 (2.8)
Ingrown hair	6 (2.8)
Basal cell carcinoma	5 (2.3)
Other sarcoma	3 (1.4)
Hemangioma or vascular malformation	3 (1.4)
Neurofibroma	3 (1.4)
Bruise	2 (0.9)
Desmoid tumor or fibromatosis	2 (0.9)
Liposarcoma	1 (0.5)
Nodular fasciitis	1 (0.5)
Other	24 (11.2)
Tumor-related clinic visits before biopsy, No. (n = 209)	
1 or 2	114 (54.5)
3 or 4	54 (25.8)
≥5	41 (19.6)
Clinicians seen before biopsy, No. (n = 213)	
1 or 2	156 (73.2)
3 or 4	38 (17.8)
≥5	19 (8.9)

^a^ More than 1 prebiopsy suspicion could be reported because many individuals had multiple clinicians with different suspicions.

Respondents reported many tumor-related visits to a clinician before receiving a biopsy for their DFSoP, with 41 of 209 (19.6%) reporting 5 or more visits (Table). In addition, 19 of 213 respondents (8.9%) visited 5 or more clinicians before receiving a biopsy. The biopsy was requested by 51 respondents (23.8%), while 163 (76.2%) were offered a biopsy.

### Dermatofibrosarcoma, Often Protuberant

**Figure 1.**
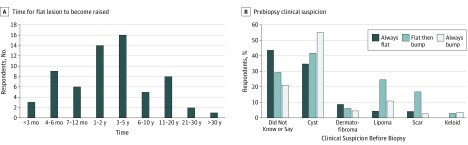
Behavior and Appearance of Flat Dermatofibrosarcoma, Often Protuberant, Lesions A, Respondents with a flat lesion that developed into a bump reported the length of time it took for the lesion to become raised. B, The clinical suspicion before biopsy varied based on whether the lesion was flat, was flat then became raised, or was always raised.

Dermatofibrosarcoma, often protuberant is often not protuberant, yet it is well known for its “protuberans” stage. However, 87 of 194 respondents (44.8%) first noticed their DFSoP as a flat plaque, while 107 of 194 (55.2%) first noticed a bump. Those who did not know the original shape of their tumor were excluded. Among the 87 lesions that began flat, 64 (73.6%) became protuberant eventually, but 23 (26.4%) remained flat. A wide time range was reported for a flat lesion to become a bump, with only 18 of 64 respondents (28.1%) developing a bump within 1 year of identifying the flat lesion (Figure 1A). The prebiopsy clinical suspicion varied if the lesion was always flat, flat then a bump, or always a bump (Figure 1B).

### Efficacy of Nonsurgical Treatments

Imatinib therapy was received by 9 of 201 respondents (4.5%): 4 of the 9 respondents (44.4%) reported that it decreased the size of the tumor; 2 of 9 (22.2%) reported that the tumor responded for a while but stopped responding; and 3 of 9 (33.3%) did not know if the tumor responded. Three recipients of imatinib therapy (33.3%) were also treated with radiotherapy.

Radiotherapy was received by 15 of 201 respondents (7.5%): 2 of the 15 respondents (13.3%) stated that radiotherapy decreased the size of the tumor, 1 of 15 (6.7%) reported that the tumor stayed the same size, 1 of 15 (6.7%) reported that the tumor continued to grow, 1 of 15 (6.7%) stated that it was too soon to tell if radiotherapy decreased the size of the tumor, and 10 of 15 (66.7%) did not know.

### FS-DFSoP, Recurrence, Metastasis, and Imaging Modalities

Fibrosarcomatous DFSoP was reported in 13 of 186 respondents (7.0%); this number excludes those who were unsure if they had received a diagnosis of FS-DFSoP. Of the 13 respondents with FS-DFSoP, 10 (76.9%) received a diagnosis of FS-DFSoP at their initial biopsy, while 3 (23.1%) received a diagnosis at a recurrence. All 3 respondents who were diagnosed at a recurrence reported that they had not received chemotherapy or radiotherapy before their FS-DFSoP diagnosis.

Overall, 18 of 201 respondents (9.0%) had a local recurrence: 13 of 173 respondents (7.5%) with conventional DFSoP and 3 of 11 respondents (27.3%) with FS-DFSoP (Fisher exact *t* test, *P* = .06; 2 respondents were excluded for inconsistent response). Although not statistically significant, the 19.8% discrepancy in recurrence rate is noteworthy.

Some individuals experienced multiple recurrences: 12 of 18 respondents (66.7%) with a recurrence reported 1 recurrence, 5 of 18 (27.8%) reported 2 recurrences, and 1 of 18 (5.6%) reported 5 recurrences (median follow-up time, 3 years; range, 0-21 years).

Recurrences were treated with surgery alone in 11 of 18 respondents (61.1%); surgery plus imatinib therapy and radiotherapy in 2 of 18 (11.1%); and no treatment in 1 of 18 (5.6%), and not applicable for 4 of 18 (22.2%), which may represent recurrences that have not yet been treated. Only 1 of 18 people with a recurrence (5.6%) also reported a metastasis of DFSoP (metastasis to skin).

Several DFSP FBSG members expressed concern that pain at the surgery site may indicate recurrence. Pain at the surgical site was noted by 129 of 173 respondents (74.6%), but it was associated with only 5 of 18 recurrences (27.8%), showing that pain at the surgical site was not a useful factor independently associated with recurrence. Individuals with recurrences were asked additional questions about their recurrence symptoms, and they most frequently noticed something that felt like a lump, bump, or knot (9 of 18 [50.0%]); itchiness (6 of 18 [33.3%]); and/or a sharp or stabbing pain (4 of 18 [22.2%]).

Metastasis was reported in 4 of 201 respondents (2.0%), with 2 of 173 respondents with DFSoP (1.2%) reporting metastasis and 2 of 13 respondents with FS-DFSoP (15.4%) reporting metastasis (Fisher exact test, *P* = .03). This finding should be interpreted with caution because only 4 respondents reported metastasis. Sites of metastasis included FS-DFSoP metastasic to muscle in 2 patients, DFSoP metastasis to skin in 1 patient, and DFSoP metastasic to both lung and lymph node in 1 patient. No mortalities were reported. The metastases were identified by magnetic resonance imaging (skin), radiography (1 individual with lung and lymph node metastases), and computed tomographic scan, magnetic resonance imaging, and positron emission tomographic scan (muscle; n = 1); 1 individual with muscle metastasis answered not applicable.

### Tumor Size and Delayed Diagnosis

Fifty-eight of 81 patients (71.6%) with a tumor measuring 5 cm or less received a diagnosis of DFSoP within 1 year of seeking care, whereas 33 of 59 patients (55.9%) with a tumor larger than 5 cm received a diagnosis within 1 year. However, this finding did not reach statistical significance (Fisher exact test, 2-tailed *P* = .07).

### Outcomes After Surgery

**Figure 2.**
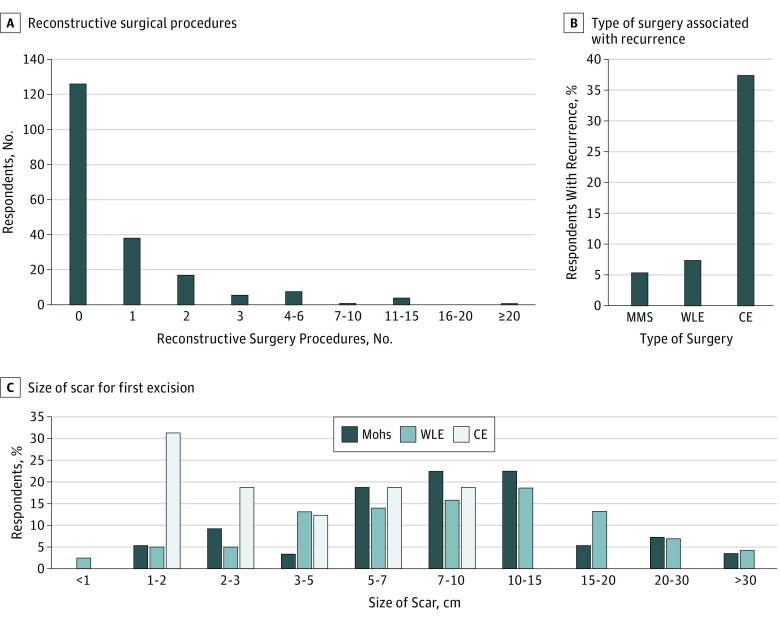
Surgical Procedures and Outcomes A, Number of reconstructive surgeries per respondent. B, The likelihood of recurrence depends on the type of surgery. C, Scar size for first excision varies based on surgery type. CE indicates conservative excision; MMS, Mohs micrographic surgery; WLE, wide local excision.

Many respondents required reconstructive surgery (75 of 201 [37.3%]) (Figure 2A). In addition, 60 of 201 respondents (29.9%) received a skin graft; 13 of these respondents (21.7%) had a skin graft that failed at some time.

Of the 194 individuals who had surgery and recalled the type of surgery, 56 (28.9%) initially underwent Mohs micrographic surgery, 122 (62.9%) had WLE, and 16 (8.2%) had a CE. Wide local excision more often resulted in a scar size of 10 cm or more (49 of 113 [43.4%]), as did Mohs micrographic surgery (21 of 53 [39.6%]), while none of the 16 CE procedures did (Figure 2C) (Fisher exact test, 2-tailed *P* = .007). When comparing WLE with Mohs micrographic surgery, there was not a significant difference in scar size (Fisher exact test, 2-tailed, *P* = .74).

Recurrence was reported in 3 of 56 Mohs micrographic surgical procedures (5.4%), 9 of 122 WLEs (7.4%), and 6 of 16 CEs (37.5%) (Figure 2B). The higher rate of recurrence with CE was statistically significant (Fisher exact test, 2-tailed *P* = .001), but there was not a significant difference between WLE and Mohs micrographic surgery (Fisher exact test, 2-tailed *P* = .76).

### Personal and Family History

One respondent of 201 (0.5%) reported a family member who also had DFSoP or FS-DFSoP. Five of 201 respondents (2.5%) were aware that they had a genetic cancer disorder: 1 *BRCA* mutation, 1 Lynch syndrome, and 3 with a genetic cancer disorder that was not a survey option.

An additional cancer diagnosis was reported by 22 of 200 respondents (11.0%): 10 of 200 respondents (5.0%) reported basal cell carcinoma, 4 of 200 (2.0%) reported melanoma, 1 of 200 (0.5%) reported uterine cancer, and 11 of 200 (5.5%) reported a cancer diagnosis that was not a survey option. More than 1 additional cancer diagnosis could be reported: 3 individuals had both melanoma and basal cell carcinoma and 1 individual had uterine cancer and an additional cancer not present on the survey. Of these 22 respondents with an additional cancer diagnosis, only 1 reported that they had a genetic cancer disorder (*BRCA* mutation). At the time of the survey, the mean (SD) age of those reporting an additional cancer diagnosis was 52.9 (8.3) years, while the mean age of all respondents to this question was 40.8 (12.1) years.

## Discussion

Facebook patient support groups can align research with patient concerns and facilitate participant recruitment. These groups are a useful method to collaborate with individuals with rare diseases. This collaboration informed the design of our study to include questions relevant to patients with DFSoP. Patient advisory board members also advocated for our study, encouraging FBSG members to participate and further DFSoP research. The FBSG and the nonprofit organization database enabled us to survey 214 respondents with a rare disease in only 3 weeks.

Patients with DFSoP perceive frequent misdiagnoses and delayed diagnosis. Previously published data suggested that DFSoP is most frequently diagnosed at age 35 to 55 years.^2^ Our study supports this finding; however, we found that patients first noticed their lesions at a much younger mean age of 29.6 years, with a median total time from first noticing the lesion to receiving a correct diagnosis of 4 years. Our study found that only 2.3% of lesions were first noticed by a clinician, and most were first noticed by the patient. We showed that most patients with DFSoP think they are misdiagnosed, with a maximum reported diagnostic delay of 41 years. Early detection of DFSoP appears to be important, as we found that more patients with a smaller tumor size received a diagnosis within 1 year of seeking care than those with a larger tumor size at excision (however, this finding did not reach statistical significance).

Dermatofibrosarcoma protuberans is a misnomer. Our respondents often initially noticed DFSoP as a flat lesion (44.8%), similar to previously published findings (43%).^7^ Given the diagnostic delays and high prevalence of flat DFSP, we suggest altering the name of dermatofibrosarcoma protuberans (DFSP) to dermatofibrosarcoma, often protuberant (DFSoP) as a reminder to clinicians that protuberant growth is not required.

The rate of DFSoP recurrence and metastasis is lower than that of FS-DFSoP. Our data show that FS-DFSoP recurred more often than conventional DFSoP (27.3% vs 7.5%), although this value did not reach statistical significance (*P* = .06). This finding compares with previously reported recurrence rates of 29.8% for FS-DFSoP and 13.7% for DFSoP.^8^

The rate of metastasis in our respondents was significantly higher in those with FS-DFSoP compared with those with DFSoP (15.4% vs 1.2%; *P* = .03, Fisher exact test), similar to published findings (14.4% vs 1.1%).^8^ However the number of patients reporting metastasis in our study was small (n = 4).

Wide local excision and Mohs micrographic surgery yielded lower local recurrence rate and larger scars compared with CE. Both WLE and Mohs micrographic surgery were significantly associated with a scar size larger than 10 cm compared with CE. Because the goal of CE is usually to remove a bulky tumor from a sensitive site such as the groin or face, but not necessarily to achieve negative margins, it is not surprising to find a smaller scar size for CE. We do not have all the clinical data behind each case, so we do not know why certain respondents underwent WLE vs Mohs micrographic surgery. Conservative excision had a significantly higher rate of tumor recurrence (37.5%) compared with WLE (7.4%) and Mohs micrographic surgery (5.4%), in line with previously published recurrence rates (Mohs micrographic surgery, 0%-3%; WLE, 0%-30.8%).^3,6,10,11^ However, the difference in recurrence between WLE and Mohs micrographic surgery did not reach statistical significance in our study. Although the recurrence rate after Mohs micrographic surgery in our study (3 of 56 [5.4%]) is higher than the previously reported 0% to 3% recurrence rates, the number of patients with recurrence in our study was very small. This finding likely represents a random variation rather than a significant difference from previous studies.

### Limitations

Our study does have several limitations. No pathologic examination reports or pathologic slides were examined to verify diagnosis, allowing for potential errors by patients completing the survey. The study was not designed with a control group. In addition to the daily FBSG survey advertisements, many PAB members sent private Facebook messages to members of their DFSP FBSG reminding them to participate in the survey, but these reminders were not tracked. Advertisements were posted only to members of private DFSP FBSGs or emailed to patients in the nonprofit organization database, so there may be a selection bias toward patients who are literate, are technologically savvy, and have internet access.

We thought that deceased patients would be represented through surviving family FBSG members. Members of the DFSoP FBSG groups are aware of several FBSG members who died from DFSoP. However, no mortalities were reported in our survey. Deceased patients and those with poor performance status may be underrepresented, creating the possibility of a fit patient selection bias. Women represented 79.0% of respondents, which is larger than the previously reported female DFSoP incidence of 53.1%.^2^ Our data likely have a selection bias toward females, skewed by the higher proportion of women using Facebook (77% of female internet users, compared with 66% of men at the time of our survey)^15^ and that women are 1.5 times more likely to complete a survey.^16^ Furthermore, we did not investigate whether there is a racial bias to our study design.

### Recommendations

It is concerning that DFSoP most often clinically mimics a cyst, lipoma, or scar, but removal of those lesions is often considered a cosmetic procedure. We recommend biopsy or removal of lesions in the following scenarios: lesions resembling a cyst but lacking a punctum or history of draining malodorous material, lesions resembling a scar but lacking a history of previous surgery or trauma, and lesions resembling a cyst, scar, or lipoma that are larger than 2.5 cm, are increasing in size over time, or are concerning to the patient. Patients should be counseled that any change in pain, color, size, or shape of their lesion should prompt expedient reevaluation by a clinician; lesions suspicious for sarcoma are ideally referred directly to a comprehensive cancer center. Biopsy with microscopic evaluation of tissue is the only way to be sure of the diagnosis of cyst, lipoma, or scar vs DFSoP or other malignant neoplasms.

## Conclusions

The findings suggest that diagnostic delays for patients with DFSoP are prevalent. Lesions were often first noticed by the patient as flat and were on average first discovered by patients in their 20s, prompting a different perspective on a tumor previously defined as protuberant and associated with a slightly older population. Dermatofibrosarcoma, often protuberant may clinically resemble benign cysts, lipomas, dermatofibromas, scars, or keloids, which is problematic because excision of these mimics is often not prioritized or recommended. Our study did not show a significant difference between outcomes with Mohs micrographic surgery or WLE, although both modalities had fewer recurrences than with CE. However, individual patient circumstances may make CE the only feasible option. We have also shown that FBSGs appear to be powerful tools to synergize effective and rapid research collaborations with large numbers of international patients with rare disease.

## Notes

Supplement. **eAppendix.** DFSP Survey–1
