# Supplementary material for: Perspectives of Patients With Dermatofibrosarcoma Protuberans on Diagnostic Delays, Surgical Outcomes, and Nonprotuberance
Source: JAMA Netw Open. 2019 Aug 30;2(8):e1910413. doi: 10.1001/jamanetworkopen.2019.10413 (PMC6724159; doi:10.1001/jamanetworkopen.2019.10413)
Supplement: Supplement. — eAppendix. DFSP Survey–1 [file jamanetwopen-2-e1910413-s001.pdf]

Supplementary Online Content

David MP, Funderberg A, Selig JP, et al. Perspectives of patients with dermatofibrosarcoma protuberans on diagnostic delays, surgical outcomes, and nonprotuberance. *JAMA Netw Open.* 2019;2(8):e1910413. doi:10.1001/jamanetworkopen.2019.10413

eAppendix. DFSP Survey–1

This supplementary material has been provided by the authors to give readers additional information about their work.

© 2019 David MP et al. *JAMA Network Open.*

eAppendix. DFSP Survey - 1

Dermatofibrosarcoma Protuberans (DFSP) Survey

THERE WILL BE TWO SURVEYS. THE SECOND SURVEY WILL BEGIN AUTOMATICALLY AFTER YOU SUBMIT THE FIRST SURVEY. YOU MUST COMPLETE BOTH SURVEYS.

Before starting the survey, you may find it helpful to get your pathology report and a ruler with centimeter (cm) markings.

This survey may take up to an hour to complete. If you are unable to complete the survey in one session, you may save your answers and return later to finish. To do so, you will need to provide your e-mail address and a unique password for the software to identify you and return you to your survey responses.

Your e-mail addresses will only be stored in the software for purposes of identification should you choose to complete the survey in more than one session. Your identification will remain anonymous to the researchers.

DFSP= Dermatofibrosarcoma Protuberans

GCF= Giant Cell Fibroblastoma

FS-DFSP= Fibrosarcomatous Dermatofibrosarcoma Protuberans

"Diagnosis," "condition," and "tumour" all refer to DFSP, GCF and/or FS-DFSP

"Provider," and "healthcare provider" all refer to medical professionals such as doctors, primary care physicians, general practioners, nurse practioners and physician assistants.

There are 70 questions in this survey

Demographics

2 [Q1]Are you responding to these questions on behalf of another individual who has/had DFSP or GCF? *

Please choose **only one** of the following:

- No
- Yes, I am responding for my child
- Yes, I am responding for my parent
- yes, i am responding for my spouse
- Yes, I am responding for my sibling
- Yes, I am responding for another relative or friend

3 [Q2]Were you born male or female? (Please answer this question with your sex, not your gender) *

Please choose **only one** of the following:

- male
- female

© 2019 David MP et al. *JAMA Network Open.*

4 [Q3]What is your race? *

Please choose **only one** of the following:

- ○ American Indian or Alaskan Native
- ○ Hispanic or Latino
- ○ Asian
- ○ Black or African
- ○ Native Hawaiian or Pacific Islander
- ○ White or Caucasian
- ○ Aborigine
- ○ More than one race
- ○ Other/Unknown/Choose not to answer

© 2019 David MP et al. *JAMA Network Open.*

5 [Q4]

What year were you born? *

Please choose **only one** of the following:

- 1915
- 1916
- 1917
- 1918
- 1919
- 1920
- 1921
- 1922
- 1923
- 1924
- 1925
- 1926
- 1927
- 1928
- 1929
- 1930
- 1931
- 1932
- 1933
- 1934
- 1935
- 1936
- 1937
- 1938
- 1939
- 1940
- 1941
- 1942
- 1943
- 1944
- 1945
- 1946
- 1947
- 1948
- 1949

© 2019 David MP et al. *JAMA Network Open.*

- ◯ 1950
- ◯ 1951
- ◯ 1952
- ◯ 1953
- ◯ 1954
- ◯ 1955
- ◯ 1956
- ◯ 1957
- ◯ 1958
- ◯ 1959
- ◯ 1960
- ◯ 1961
- ◯ 1962
- ◯ 1963
- ◯ 1964
- ◯ 1965
- ◯ 1966
- ◯ 1967
- ◯ 1968
- ◯ 1969
- ◯ 1970
- ◯ 1971
- ◯ 1972
- ◯ 1973
- ◯ 1974
- ◯ 1975
- ◯ 1976
- ◯ 1977
- ◯ 1978
- ◯ 1979
- ◯ 1980
- ◯ 1981
- ◯ 1982
- ◯ 1983
- ◯ 1984
- ◯ 1985
- ◯ 1986
- ◯ 1987
- ◯ 1988

© 2019 David MP et al. *JAMA Network Open.*

- ◯ 1989
- ◯ 1990
- ◯ 1991
- ◯ 1992
- ◯ 1993
- ◯ 1994
- ◯ 1995
- ◯ 1996
- ◯ 1997
- ◯ 1998
- ◯ 1999
- ◯ 2000
- ◯ 2001
- ◯ 2002
- ◯ 2003
- ◯ 2004
- ◯ 2005
- ◯ 2006
- ◯ 2007
- ◯ 2008
- ◯ 2009
- ◯ 2010
- ◯ 2011
- ◯ 2012
- ◯ 2013
- ◯ 2014
- ◯ 2015

© 2019 David MP et al. *JAMA Network Open.*

6 [Q5]

What year was it when something unusual first appeared in the location of your tumour? *

Please choose **only one** of the following:

- ◯ 1915
- ◯ 1916
- ◯ 1917
- ◯ 1918
- ◯ 1919
- ◯ 1920
- ◯ 1921
- ◯ 1922
- ◯ 1923
- ◯ 1924
- ◯ 1925
- ◯ 1926
- ◯ 1927
- ◯ 1928
- ◯ 1929
- ◯ 1930
- ◯ 1931
- ◯ 1932
- ◯ 1933
- ◯ 1934
- ◯ 1935
- ◯ 1936
- ◯ 1937
- ◯ 1938
- ◯ 1939
- ◯ 1940
- ◯ 1941
- ◯ 1942
- ◯ 1943
- ◯ 1944
- ◯ 1945
- ◯ 1946
- ◯ 1947
- ◯ 1948

© 2019 David MP et al. *JAMA Network Open.*

- 1949
- 1950
- 1951
- 1952
- 1953
- 1954
- 1955
- 1956
- 1957
- 1958
- 1959
- 1960
- 1961
- 1962
- 1963
- 1964
- 1965
- 1966
- 1967
- 1968
- 1969
- 1970
- 1971
- 1972
- 1973
- 1974
- 1975
- 1976
- 1977
- 1978
- 1979
- 1980
- 1981
- 1982
- 1983
- 1984
- 1985
- 1986
- 1987

© 2019 David MP et al. *JAMA Network Open.*

- ◯ 1988
- ◯ 1989
- ◯ 1990
- ◯ 1991
- ◯ 1992
- ◯ 1993
- ◯ 1994
- ◯ 1995
- ◯ 1996
- ◯ 1997
- ◯ 1998
- ◯ 1999
- ◯ 2000
- ◯ 2001
- ◯ 2002
- ◯ 2003
- ◯ 2004
- ◯ 2005
- ◯ 2006
- ◯ 2007
- ◯ 2008
- ◯ 2009
- ◯ 2010
- ◯ 2011
- ◯ 2012
- ◯ 2013
- ◯ 2014
- ◯ 2015

© 2019 David MP et al. *JAMA Network Open.*

7 [Q6]What year did you first see a healthcare provider for your tumour? *

Please choose **only one** of the following:

- 1915
- 1916
- 1917
- 1918
- 1919
- 1920
- 1921
- 1922
- 1923
- 1924
- 1925
- 1926
- 1927
- 1928
- 1929
- 1930
- 1931
- 1932
- 1933
- 1934
- 1935
- 1936
- 1937
- 1938
- 1939
- 1940
- 1941
- 1942
- 1943
- 1944
- 1945
- 1946
- 1947
- 1948
- 1949
- 1950

© 2019 David MP et al. *JAMA Network Open.*

- ◯ 1951
- ◯ 1952
- ◯ 1953
- ◯ 1954
- ◯ 1955
- ◯ 1956
- ◯ 1957
- ◯ 1958
- ◯ 1959
- ◯ 1960
- ◯ 1961
- ◯ 1962
- ◯ 1963
- ◯ 1964
- ◯ 1965
- ◯ 1966
- ◯ 1967
- ◯ 1968
- ◯ 1969
- ◯ 1970
- ◯ 1971
- ◯ 1972
- ◯ 1973
- ◯ 1974
- ◯ 1975
- ◯ 1976
- ◯ 1977
- ◯ 1978
- ◯ 1979
- ◯ 1980
- ◯ 1981
- ◯ 1982
- ◯ 1983
- ◯ 1984
- ◯ 1985
- ◯ 1986
- ◯ 1987
- ◯ 1988
- ◯ 1989

© 2019 David MP et al. *JAMA Network Open.*

- ○ 1990
- ○ 1991
- ○ 1992
- ○ 1993
- ○ 1994
- ○ 1995
- ○ 1996
- ○ 1997
- ○ 1998
- ○ 1999
- ○ 2000
- ○ 2001
- ○ 2002
- ○ 2003
- ○ 2004
- ○ 2005
- ○ 2006
- ○ 2007
- ○ 2008
- ○ 2009
- ○ 2010
- ○ 2011
- ○ 2012
- ○ 2013
- ○ 2014
- ○ 2015

© 2019 David MP et al. *JAMA Network Open.*

8 [Q7]What was the year when you were first officially diagnosed with DFSP or GCF? *

Please choose **only one** of the following:

- 1915
- 1916
- 1917
- 1918
- 1919
- 1920
- 1921
- 1922
- 1923
- 1924
- 1925
- 1926
- 1927
- 1928
- 1929
- 1930
- 1931
- 1932
- 1933
- 1934
- 1935
- 1936
- 1937
- 1938
- 1939
- 1940
- 1941
- 1942
- 1943
- 1944
- 1945
- 1946
- 1947
- 1948
- 1949

© 2019 David MP et al. *JAMA Network Open.*

- ◯ 1950
- ◯ 1951
- ◯ 1952
- ◯ 1953
- ◯ 1954
- ◯ 1955
- ◯ 1956
- ◯ 1957
- ◯ 1958
- ◯ 1959
- ◯ 1960
- ◯ 1961
- ◯ 1962
- ◯ 1963
- ◯ 1964
- ◯ 1965
- ◯ 1966
- ◯ 1967
- ◯ 1968
- ◯ 1969
- ◯ 1970
- ◯ 1971
- ◯ 1972
- ◯ 1973
- ◯ 1974
- ◯ 1975
- ◯ 1976
- ◯ 1977
- ◯ 1978
- ◯ 1979
- ◯ 1980
- ◯ 1981
- ◯ 1982
- ◯ 1983
- ◯ 1984
- ◯ 1985
- ◯ 1986
- ◯ 1987
- ◯ 1988

© 2019 David MP et al. *JAMA Network Open.*

- ◯ 1989
- ◯ 1990
- ◯ 1991
- ◯ 1992
- ◯ 1993
- ◯ 1994
- ◯ 1995
- ◯ 1996
- ◯ 1997
- ◯ 1998
- ◯ 1999
- ◯ 2000
- ◯ 2001
- ◯ 2002
- ◯ 2003
- ◯ 2004
- ◯ 2005
- ◯ 2006
- ◯ 2007
- ◯ 2008
- ◯ 2009
- ◯ 2010
- ◯ 2011
- ◯ 2012
- ◯ 2013
- ◯ 2014
- ◯ 2015

© 2019 David MP et al. *JAMA Network Open.*

9 [Q8]

What was the year when you had your first excision? *

Please choose **only one** of the following:

- 1915
- 1916
- 1917
- 1918
- 1919
- 1920
- 1921
- 1922
- 1923
- 1924
- 1925
- 1926
- 1927
- 1928
- 1929
- 1930
- 1931
- 1932
- 1933
- 1934
- 1935
- 1936
- 1937
- 1938
- 1939
- 1940
- 1941
- 1942
- 1943
- 1944
- 1945
- 1946
- 1947
- 1948
- 1949

© 2019 David MP et al. *JAMA Network Open.*

- ○ 1950
- ○ 1951
- ○ 1952
- ○ 1953
- ○ 1954
- ○ 1955
- ○ 1956
- ○ 1957
- ○ 1958
- ○ 1959
- ○ 1960
- ○ 1961
- ○ 1962
- ○ 1963
- ○ 1964
- ○ 1965
- ○ 1966
- ○ 1967
- ○ 1968
- ○ 1969
- ○ 1970
- ○ 1971
- ○ 1972
- ○ 1973
- ○ 1974
- ○ 1975
- ○ 1976
- ○ 1977
- ○ 1978
- ○ 1979
- ○ 1980
- ○ 1981
- ○ 1982
- ○ 1983
- ○ 1984
- ○ 1985
- ○ 1986
- ○ 1987
- ○ 1988

© 2019 David MP et al. *JAMA Network Open.*

- ◯ 1989
- ◯ 1990
- ◯ 1991
- ◯ 1992
- ◯ 1993
- ◯ 1994
- ◯ 1995
- ◯ 1996
- ◯ 1997
- ◯ 1998
- ◯ 1999
- ◯ 2000
- ◯ 2001
- ◯ 2002
- ◯ 2003
- ◯ 2004
- ◯ 2005
- ◯ 2006
- ◯ 2007
- ◯ 2008
- ◯ 2009
- ◯ 2010
- ◯ 2011
- ◯ 2012
- ◯ 2013
- ◯ 2014
- ◯ 2015

© 2019 David MP et al. *JAMA Network Open.*

Symptom Burden

10 [Q8]I would describe the severity of my symptoms to be: *

Please choose **only one** of the following:

- I do not have symptoms any longer
- Mild
- Problematic but manageable
- Problematic but manageable half the time
- Unmanageable over half the time
- Severe, unmanageable most of the time

11 [Q9]I would describe the burden of my DFSP or GCF related physical symptoms as: *

Please choose **only one** of the following:

- I do not have symptoms any longer
- Mild
- Moderate
- Severe

12 [Q10]How many doctors did you visit before finding one that understood how to treat your sarcoma? *

Please choose **only one** of the following:

- 1-2
- 3-4
- 5-6
- 7-8
- 9-10
- More than 10

© 2019 David MP et al. *JAMA Network Open.*

13 [Q11]The doctor who FIRST treated my tumour related symptoms had a level of understanding of my diagnosis that could be described as: *

Please choose **only one** of the following:

- ○ Poor or no understanding
- ○ Minimal understanding
- ○ Acceptable level of understanding
- ○ A high level of understanding
- ○ The highest level of understanding

14 [Q12]My CURRENT healthcare provider's level of understanding about treating and managing my condition could be described as: *

Please choose **only one** of the following:

- ○ Poor or not understanding
- ○ Minimal understanding
- ○ Acceptable level of understanding
- ○ A high level of understanding
- ○ The highest level of understanding
- ○ I am not currently under a provider's care for my DFSP or GCF

15 [Q13]My CURRENT healthcare provider's understanding of my condition is: *

Please choose **only one** of the following:

- ○ Poor or no understanding
- ○ Minimal understanding
- ○ Acceptable level of understanding
- ○ A high level of understanding
- ○ The highest level of understanding
- ○ I am not currently under a provider's care for my DFSP or GCF

© 2019 David MP et al. *JAMA Network Open.*

16 [Q14]My CURRENT healthcare provider's understanding of treatment options for my condition is: *

Please choose **only one** of the following:

- Poor or minimal understanding
- Minimal understanding
- Acceptable level of understanding
- A high level of understanding
- The highest level of understanding
- I am not currently under a provider’s care for my DFSP or GCF

17 [Q15]My CURRENT healthcare provider's level of understanding of my risk of recurrence and/or future metastasis is: (Metastases= tumour spread to another part of your body like brain, lungs, or other internal organs. Recurrence= tumour growing back at the SAME location where it was first found) *

Please choose **only one** of the following:

- Poor or no understanding
- Minimal understanding
- Acceptable level of understanding
- A high level of understanding
- The highest level of understanding
- I am not currently under a provider’s care for my DFSP or GCF

18 [Q16]My CURRENT healthcare provider's level of understanding of how my symptoms could progress is: *

Please choose **only one** of the following:

- Poor or no understanding
- Minimal understanding
- Acceptable level of understanding
- A high level of understanding
- The highest level of understanding
- I am not currently under a provider’s care for my DFSP or GCF

© 2019 David MP et al. *JAMA Network Open.*

19 [Q17]My CURRENT healthcare provider's level of understanding of how DFSP or GCF influences my future risk of other types of cancers is: *

Please choose **only one** of the following:

- Poor or no understanding
- Minimal understanding
- Acceptable level of understanding
- A high level of understanding
- The highest level of understanding
- I am not currently under a provider's care for my DFSP or GCF

20 [Q18]When trying to find information about my diagnosis, my satisfaction with the materials available is: *

Please choose **only one** of the following:

- Very dissatisfied
- Somewhat dissatisfied
- Neither satisfied nor dissatisfied
- Somewhat satisfied
- Very satisfied

21 [Q19]I can find information about my diagnosis when I look for it in the library, online or other places: *

Please choose **only one** of the following:

- Strongly disagree
- Somewhat disagree
- Neither disagree nor agree
- Somewhat agree
- Strongly agree

© 2019 David MP et al. *JAMA Network Open.*

22 [Q20]The time and effort it takes me to find information about my diagnosis is: *

Please choose **only one** of the following:

- Unacceptable, burdensome
- Barely acceptable to unacceptable some of the time
- Acceptable
- Challenging but acceptable
- Reasonable

23 [Q21]The quality of information I can find regarding my diagnosis is: *

Please choose **only one** of the following:

- Poor
- Below average
- Average
- Above average
- Excellent

24 [Q22]It is challenging to find information regarding treatment options for my tumour: *

Please choose **only one** of the following:

- Strongly agree
- Somewhat agree
- Neither agree nor disagree
- Somewhat disagree
- Strongly disagree

25 [Q23]The length of time between seeking help for my tumour from a healthcare provider and being tested for DFSP or GCF decreased the amount of trust I have in my healthcare provider. *

Please choose **only one** of the following:

- Strongly agree
- Somewhat agree
- Neither agree nor disagree
- Somewhat disagree
- Strongly disagree

© 2019 David MP et al. *JAMA Network Open.*

26 [Q24]Delayed actions in testing for cancer negatively affects my opinion of my provider's ability to treat my condition: *

Please choose **only one** of the following:

- Strongly agree
- Somewhat agree
- Neither agree nor disagree
- Somewhat disagree
- Strongly disagree

27 [Q25]The delay in testing for cancer influenced the severity of the impact of DFSP or GCF as I experience it today: *

Please choose **only one** of the following:

- Strongly agree
- Somewhat agree
- Neither agree nor disagree
- Somewhat disagree
- Strongly disagree

28 [Q26]Earlier testing could have helped with the severity of my cancer as I experience it today: *

Please choose **only one** of the following:

- Strongly agree
- Somewhat agree
- Neither agree nor disagree
- Somewhat disagree
- Strongly disagree

© 2019 David MP et al. *JAMA Network Open.*

29 [Q27]The delay in testing had a negative effect on my opinion of all healthcare providers: *

Please choose **only one** of the following:

- Strongly agree
- Somewhat agree
- Neither agree nor disagree
- Somewhat disagree
- Strongly disagree

30 [Q28]The delay in cancer testing increased the level of responsibility I assume for managing my health: *

Please choose **only one** of the following:

- Strongly agree
- Somewhat agree
- Neither agree nor disagree
- Somewhat disagree
- Strongly disagree

31 [Q29]The delay in cancer testing decreased my trust in ALL healthcare providers: *

Please choose **only one** of the following:

- Strongly agree
- Somewhat agree
- Neither agree nor disagree
- Somewhat disagree
- Strongly disagree

© 2019 David MP et al. *JAMA Network Open.*

32 [Q30]

If you have interviewed for a job since your DFSP or GCF diagnosis, did you tell your potential employers about your diagnosis? *

Please choose **only one** of the following:

- ◯ Yes
- ◯ At some interviews I did and at some I did not
- ◯ No
- ◯ I have not interviewed for a job since I was diagnosed

33 [Q31]

Were you offered jobs at interviews where you told potential employers about your diagnosis? *

Only answer this question if the following conditions are met:
° ((**Q30.NAOK** == "1" or **Q30.NAOK** == "2"))

Please choose **only one** of the following:

- ◯ Yes
- ◯ No
- ◯ I was offered some jobs, but not others

34 [Q32]Did you experience any of these problems because of your DIAGNOSIS? *

Please choose **all** that apply:

- ☐ Depression
- ☐ Anxiety
- ☐ Isolation
- ☐ Sleep problems
- ☐ Negative body image
- ☐ Obsessing about recurrences or metastasis
- ☐ Other not mentioned here
- ☐ I did not experience any of these

© 2019 David MP et al. *JAMA Network Open.*

35 [Q33]

Did you experience any of these problems because of your SURGICAL treatment(s)? *

Please choose **all** that apply:

- ☐ Depression
- ☐ Anxiety
- ☐ Isolation
- ☐ Sleep problems
- ☐ Negative body image
- ☐ Post-traumatic stress disorder
- ☐ Survivor's guilt
- ☐ Other not mentioned here
- ☐ I did not experience any of these
- ☐ Not applicable

36 [Q34]Were you given any information on DFSP or GCF by your doctor when you were first given your diagnosis? *

Please choose **only one** of the following:

- ○ Yes, I was given printed information
- ○ Yes, I was given verbal information
- ○ No, I was not given any information

37 [Q35]How did you find information about DFSP or GCF? *

Please choose **all** that apply:

- ☐ Facebook
- ☐ Medical websites
- ☐ Medical professionals (doctors, nurses, etc.)
- ☐ Books
- ☐ Other

© 2019 David MP et al. *JAMA Network Open.*

Prior to diagnosis

These questions are about the time period before you went to a healthcare provider for your tumour (which may have been DFSP, GCF, or FS-DFSP)

38 [Q36]Did your tumour occur at the location of...? *

Please choose **only one** of the following:

- A tattoo
- A previous vaccination
- A burn
- A scar NOT from surgery for a previous growth at that site
- A scar that IS from a surgery for a previous growth at that site
- A previous injory or trauma
- Nothing was present at that site before the DFSP/GCF occurred
- An insect/animal bite
- I do not know/I do not remember

39 [Q37]How much time passed between when you had your tattoo/vaccination/burn/scar/previous injury or trauma and when you noticed the tumour at the same location? *

Only answer this question if the following conditions are met:
° ((**Q36.NAOK** == "1" or **Q36.NAOK** == "2" or **Q36.NAOK** == "3" or **Q36.NAOK** == "4" or **Q36.NAOK** == "5" or **Q36.NAOK** == "6"))

Please choose **only one** of the following:

- 3 months or less
- 3-6 months
- 7 months-1year
- 1 year-2 years
- 3-5 years
- 6-10 years
- 11-20 years
- 21-30 years
- Over 30 years

© 2019 David MP et al. *JAMA Network Open.*

40 [Q38]Where on your body was the tumour located? *

Please choose **all** that apply:

- ☐ Scalp
- ☐ Forehead
- ☐ Ear
- ☐ Face
- ☐ Neck
- ☐ Shoulders
- ☐ Upper back
- ☐ Lower back
- ☐ Breast/Chest
- ☐ Abdomen
- ☐ Upper arm (above elbows)
- ☐ Lower arm (below elbows)
- ☐ Hand or finger
- ☐ Upper leg (above knees)
- ☐ Lower leg (below knees)
- ☐ Foot or toe
- ☐ Buttocks
- ☐ Anus
- ☐ Male genitals (penis or scrotum)
- ☐ Female genitals (vulva)
- ☐ Mons (the hairy pubic area above the female genitals)

© 2019 David MP et al. *JAMA Network Open.*

41 [Q39]

When you first noticed the tumour, were you on any hormonal medication? (For example, birth control pills, progesterone, estrogen, corticosteroids)? *

Please choose **only one** of the following:

- No
- Yes, testosterone
- Yes, birth control hormonal medication
- Yes tamoxifen
- Yes, another (not for birth control) progesterone or progesterone-related medication
- Yes, another (not tamoxifen or for birth control) estrogen or estrogen-related medication
- Yes, corticosteroids (dexamethasone, hydrocortisone, prednisone, for example)
- Yes, other not listed here
- I do not know or do not remember

42 [Q40]Were you pregnant when you first noticed the tumour? *

Only answer this question if the following conditions are met:
° ((**Q2.NAOK** == "2"))

Please choose **only one** of the following:

- I have never been pregnant
- Yes
- No

43 [Q41]Were you breast-feeding when you first noticed the tumour? *

Only answer this question if the following conditions are met:
° ((**Q2.NAOK** == "2"))

Please choose **only one** of the following:

- I have never breast-fed an infant/child
- Yes
- No

© 2019 David MP et al. *JAMA Network Open.*

44 [Q40]

Had you STOPPED taking any hormonal medication within THREE MONTHS of when you first noticed the tumour? (For example, birth control pills, progesterone, estrogen, corticosteroids)?

Please choose **only one** of the following:

- No
- Yes, testosterone
- Yes, birth control hormonal medication
- Yes tamoxifen
- Yes, another (not for birth control) progesterone or progesterone-related medication
- Yes, another (not tamoxifen or for birth control) estrogen or estrogen-related medication
- Yes, corticosteroids (dexamethasone, hydrocortisone, prednisone, for example)
- Yes, other not listed here
- I do not know or do not remember

© 2019 David MP et al. *JAMA Network Open.*

Your diagnosis experience

45 [Q42]Who first noticed your tumour? *

Please choose **only one** of the following:

- I first noticed the tumour
- A friend/partner/family member/co-worker first noticed the tumour
- My health care provider noticed the tumour
- Imaging studies detected the tumour (such as mammogram/x-ray, CT, MRI, PET scan, ultrasound, or other studies)

46 [Q43]Describe the physical sensations of your tumour when you first went to a healthcare provider for your tumour. *

Please choose **only one** of the following:

- My sensations on the area of the tumour did not change from how normal skin feels
- The tumour was numb/had no sensation
- The tumour had a tingling sensation
- The tumour was itchy
- The tumour had a dull painful/bruise-like feeling
- The tumour had a sharp/stabbing pain
- The tumour had a burning pain
- The tumour was tender to the touch
- Other sensation not listed here
- I do not know/I do not remember

© 2019 David MP et al. *JAMA Network Open.*

47 [Q44]Describe the color of your tumour when you first went to a healthcare provider for your tumour. *

Please choose **only one** of the following:

- ◯ The tumour had the coloration of normal skin
- ◯ The tumour had a blue to purple discoloration
- ◯ The tumour had a black/brown discoloration
- ◯ The tumour had a red discoloration
- ◯ The tumour had a pink discoloration
- ◯ The tumour had a white/lighter than normal skin discoloration
- ◯ The tumour was ulcerating/had an open sore on the surface
- ◯ Other color not listed here

48 [Q45]Describe the shape of your tumour when you first went to a healthcare provider for your tumour. *

Please choose **only one** of the following:

- ◯ The tumour did NOT raise the surface of my skin (it was flat) and felt like normal tissue
- ◯ The tumour did NOT raise the surface of my skin and felt like a thickened area beneath the skin
- ◯ The tumour did NOT raise the surface of my skin and felt like a lump that I could move around (it was mobile)
- ◯ The tumour did NOT raise the surface of my skin and felt like a lump that I could NOT move around (it was not mobile)
- ◯ The tumour raised the surface of the skin in a single smooth lump/bump/knot that I could move around (it was mobile)
- ◯ The tumour raised the surface of the skin in a single smooth lump/bump/knot that I could NOT move around (it was not mobile)
- ◯ The tumour raised the surface of the skin in a complicated lumpy/bumpy growth that I could move around (it was mobile)
- ◯ The tumour raised the surface of the skin in a complicated lumpy/bumpy growth that I could NOT move around (it was not mobile)
- ◯ The tumour was made of multiple bumps/nodules

© 2019 David MP et al. *JAMA Network Open.*

49 [Q46]Before your biopsy, did you ever notice something unusual that was flat but did not raise the skin? *

Please choose **only one** of the following:

- Yes, the tumour was always flat before biopsy
- Yes, there was something unusual that started out flat and then grew into a bump
- No, I first noticed it as a bump
- Not applicable/I do not know/do not remember

50 [Q47]How long did it take for the lesion to become raised after you first noticed a change? *

Only answer this question if the following conditions are met:
° ((**Q46.NAOK** == "2"))

Please choose **only one** of the following:

- Less than 3 months after I first noticed the tumour
- Between4-6 months
- Between 7 months-1 year
- Between 1 year-2 years
- Between 3 years- 5 years
- Between 6-10 years
- Between 11-20 years
- Between 21-30 years
- Over 30 years

51 [Q48]How many tumour-related visits did you have to a healthcare provider before you tumour was biopsied? *

Please choose **only one** of the following:

- 1 visit
- 2 visits
- 3 visits
- 4 visits
- 5 visits or more
- I do not know/I do not remember

© 2019 David MP et al. *JAMA Network Open.*

52 [Q49]How many different healthcare providers did you visit before you received a biopsy? *

Please choose **only one** of the following:

- 1 healthcare provider
- 2 healthcare providers
- 3 healthcare providers
- 4 healthcare providers
- 5 or more healthcare providers
- I do not know/I do not remember

53 [Q50]Did you ask your healthcare provider for a biopsy, or was a biopsy offered to you? *

Please choose **only one** of the following:

- I had to ask for a biopsy
- My healthcare provider offered a biopsy

54 [Q51]Do you think your DFSP or GCF was ever misdiagnosed? *

Please choose **only one** of the following:

- Yes
- No
- Unsure

© 2019 David MP et al. *JAMA Network Open.*

55 [Q52]

How long did your misdiagnosis delay treatment? *

Only answer this question if the following conditions are met:
° ((**Q51.NAOK** == "1"))

Please choose **only one** of the following:

- 3 months or less
- 4-6 months
- 7 months-1 year
- Between 1-2 years
- Between 2-5 years
- Between 5-10 years
- Between 10-20 years
- Between 20-30 years
- Over 30 years

56 [Q53]

Were you given a wrong diagnosis before biopsy or after biopsy or both? *

Only answer this question if the following conditions are met:
° ((**Q51.NAOK** == "1"))

Please choose **only one** of the following:

- I was misdiagnosed before biopsy
- I was misdiagnosed after biopsy
- I was misdiagnosed both before and after biopsy

57 [Q54]Who misdiagnosed you before you had a biopsy? *

Only answer this question if the following conditions are met:
° ((**Q51.NAOK** == "1") and (**Q53.NAOK** == "1" or **Q53.NAOK** == "3"))

Please choose **all** that apply:

- Primary care provider
- Dermatologist
- Other physician
- Not applicable

© 2019 David MP et al. *JAMA Network Open.*

58 [Q55]Before your biopsy, what did your healthcare provider tell you the tumour was? *

Please choose **all** that apply:

- ☐ Angioma/hemangioma/vascular malformation
- ☐ Angiosarcoma
- ☐ Atrophoderma
- ☐ Basal cell carcinoma
- ☐ Birthmark
- ☐ Bruise
- ☐ Cyst/Epidermal (sebaceous) cyst
- ☐ Dermatofibroma/benign fibrous histiocytoma
- ☐ Desmoid tumor/fibromatosis
- ☐ Fibrosarcoma/fibromyxosarcoma
- ☐ Ingrown hair
- ☐ Kaposi sarcoma
- ☐ Keloid
- ☐ Leiomyoma
- ☐ Leiomyosarcoma
- ☐ Lipoatrophy
- ☐ Lipoma
- ☐ Liposarcoma
- ☐ Melanoma
- ☐ Morpheoa/morphea/scleroderma
- ☐ Neurofibroma
- ☐ Nodular fasciitis
- ☐ Sarcoidosis
- ☐ Scar
- ☐ Undifferentiated/unclassified soft tissue sarcoma/malignant fibrous histiocytoma/undifferentiated pleomorphic sarcoma
- ☐ Other
- ☐ I do not know/I do not remember
- ☐ My healthcare provider did not say what the tumor was before my biopsy/My healthcare provider did not know

© 2019 David MP et al. *JAMA Network Open.*

59 [Q56]What type of healthcare provider performed the first biopsy of your tumour? *

Please choose **only one** of the following:

- Primary care physician/general practitioner
- Nurse practitioner
- Physician's assistant
- Dermatologist
- Plastic surgeon
- Other surgeon
- Other
- I do not know/I do not remember

60 [Q57]Do you believe or were you told that you received a wrong diagnosis on your pathology report at any time (the report you received after your biopsy)? *

Please choose **only one** of the following:

- Yes
- No
- I do not know

© 2019 David MP et al. *JAMA Network Open.*

61 [Q58]If you believe you received a wrong diagnosis on your pathology report, what diagnosis did you receive? *

Only answer this question if the following conditions are met:
° ((**Q57.NAOK** == "1"))

Please choose **all** that apply:

- ☐ Dermatofibroma/benign fibrous histiocytoma
- ☐ Desmoid tumor/fibromatosis
- ☐ Fibrosarcoma/fibromyxosarcoma
- ☐ Leiomyosarcoma
- ☐ Undifferentiated/unclassified soft tissue sarcoma/malignant fibrous histiocytoma/undifferentiated pleomorphic sarcoma
- ☐ Atypical fibroxanthoma
- ☐ Lipoma
- ☐ Nodular fasciitis
- ☐ Scar
- ☐ Neurofibroma
- ☐ Spindle cell neoplasm/spindle cell lesion
- ☐ Nevus sebaceous/sebaceous nevus
- ☐ Leiomyoma
- ☐ Other
- ☐ I do not know/I do not remember

62 [Q59]After your first biopsy, did the tumour seem to grow more quickly than before? *

Please choose **only one** of the following:

- ○ Yes
- ○ No
- ○ Not applicable
- ○ I do not know or do not remember

© 2019 David MP et al. *JAMA Network Open.*

Your DFSP or GCF or FS-DFSP diagnosis details

Metastases= tumour spread to other part of your body like brain, lungs, or other internal organs

Recurrence= tumour growing back at the same location where it was first found

63 [Q60]When you were first diagnosed, did the tumour directly invade muscle, bone or any organs? *

Please choose **only one** of the following:

- Yes
- No, it only invaded the skin/fat/subcutis
- I do not know or do not remember

64 [Q61]About how large was the tumour when you first went to a healthcare provider? *

Please choose **only one** of the following:

- Less than 1 cm
- Between 1 cm-2 cm
- Between 2 cm-3 cm
- Between 3 cm-5 cm
- Between 5 cm-7 cm
- Between 7 cm-10 cm
- Between 10 cm-15 cm
- Between 15 cm-20 cm
- Between 20 cm-30 cm
- Over 30 cm
- I do not know or do not remember

© 2019 David MP et al. *JAMA Network Open.*

65 [Q62]About how large was your tumour on the skin surface when you were first officially diagnosed with DFSP or GCF? *

Please choose **only one** of the following:

- Less than 1 cm
- 1 cm-2 cm
- between 2 cm-3 cm
- between 3 cm-5 cm
- between 5 cm-7 cm
- between 7 cm-10 cm
- between 10 cm-15 cm
- between 15 cm-20 cm
- between 20 cm-30 cm
- Over 30 cm
- I do not know or do not remember

66 [Q63]About how large was your tumour on your pathology report when you had your first excision? *

Please choose **only one** of the following:

- Less than 1 cm
- 1cm-2 cm
- between 2 cm-3 cm
- between 3 cm-5 cm
- between 5 cm-7 cm
- between 7 cm-10 cm
- between 10 cm-15 cm
- between 15 cm-20 cm
- between 20 cm-30 cm
- Over 30 cm
- Size was not given on my pathology report
- I do not know or do not remember

© 2019 David MP et al. *JAMA Network Open.*

67 [Q64]What was the size of your scar for your first excision following your biopsy? *

Please choose **only one** of the following:

- Less than 1 cm
- 1 cm-2 cm
- between 2 cm-3 cm
- between 3 cm-5 cm
- between 5 cm-7 cm
- between 7 cm-10 cm
- between 10 cm-15 cm
- between 15 cm-20 cm
- between 20 cm-30 cm
- Over 30 cm
- I do not know or do not remember

68 [Q65]

Were there any metastases found when you were first diagnosed?

****Metastases= Tumor spread to other part of your body like brain, lungs or other internal organs ***

Please choose **only one** of the following:

- Yes
- No
- I do not know/I do not remember

© 2019 David MP et al. *JAMA Network Open.*

69 [Q66]How were the metastases found? *

Only answer this question if the following conditions are met:
° ((**Q65.NAOK** == "1"))

Please choose **all** that apply:

- ☐ CT scan
- ☐ MRI
- ☐ X-ray
- ☐ PET scan
- ☐ Ultrasound
- ☐ Other
- ☐ I do not know or do not remember

70 [Q67]When you were correctly diagnosed, which type of DFSP were you first diagnosed with? *

Please choose **only one** of the following:

- ○ DFSP
- ○ DFSP with fibrosarcomatous change (FS-DFSP)
- ○ Giant cell fibroblastoma (GCF)
- ○ Bednar tumor/pigmented DFSP /DFSP with melanin pigment
- ○ DFSP with granular cell change
- ○ Myxoid DFSP
- ○ Dermatofibrosarcoma with areas of giant cell fibroblastoma
- ○ Dermatofibrosarcoma with myoid nodules
- ○ Other
- ○ I do not know/I do not remember

© 2019 David MP et al. *JAMA Network Open.*

21.11.2015 – 00:00

Submit your survey.
Thank you for completing this survey.

© 2019 David MP et al. *JAMA Network Open.*

DFSP Survey - 2

Dermatofibrosarcoma Protuberans (DFSP) Survey

Before starting the survey, you may find it helpful to get your pathology report and a ruler with centimeter (cm) markings.

This survey may take up to an hour to complete. If you are unable to complete the survey in one session, you may save your answers and return later to finish. To do so, you will need to provide your e-mail address and a unique password for the software to identify you and return you to your survey responses.

Your e-mail addresses will only be stored in the software for purposes of identification should you choose to complete the survey in more than one session. Your identification will remain anonymous to the researchers.

DFSP= Dermatofibrosarcoma Protuberans

GCF= Giant Cell Fibroblastoma

FS-DFSP= Fibrosarcomatous Dermatofibrosarcoma Protuberans

"Diagnosis," "condition," and "tumour" all refer to DFSP, GCF and/or FS-DFSP

"Provider," and "healthcare provider" all refer to medical professionals such as doctors, primary care physicians, general practioners, nurse practioners and physician assistants.

There are 53 questions in this survey

Your first DFSP or GCF or FS-DFSP treatment

2 [Q68]Were any imaging studies of the tumour performed before your first surgery? *

Please choose **all** that apply:

- ☐ No imaging studies were performed
- ☐ An MRI of the tumour was performed
- ☐ An ultrasound of the tumour was performed
- ☐ A CT scan of the tumour was performed
- ☐ A PET scan of the tumour was performed
- ☐ An X-ray of the tumour was performed
- ☐ Photograph of lesion to follow size/medical photography/Lump map

© 2019 David MP et al. *JAMA Network Open.*

3 [Q69]When you were first diagnosed, did you have any scans or imaging of the chest to look for lung metastasis? *

Please choose **only one** of the following:

- Yes, I had a CT scan of the chest
- Yes, I had an MRI of the chest
- Yes, I had a PET scan
- Yes, I had a chest X-ray
- No

4 [Q70]What were you told when you were first diagnosed with DFSP or GCF? *

Please choose **only one** of the following:

- I was told it would not be possible or not likely possible that I could receive a complete excision due to the size of my tumour
- I was told it would not be possible or not likely possible that I could receive a complete excision due to the location of my tumour
- I was told it would not be possible or not likely possible that I could receive a complete excision due to the location AND size of my tumour
- I was told that my surgeons could attempt a complete excision
- Other

5 [Q71]What type of surgical treatment was offered to you for your DFSP or GCF at first diagnosis? *

Please choose **all** that apply:

- Wide local excision
- Mohs' micrographic surgery
- Conservative excision surgery
- Other surgery
- No surgical treatment was offered
- I do not know/I do not remember

© 2019 David MP et al. *JAMA Network Open.*

6 [Q72]When you were first diagnosed, what non-surgical treatments were offered to you? Select ALL that apply *

Please choose **all** that apply:

- ☐ Gleevec (imatinib) before surgery
- ☐ Gleevec (imatinib) after surgery
- ☐ Gleevec (imatinib) BOTH before and after surgery
- ☐ Gleevec (imatinib) and no surgery
- ☐ Radiation therapy before surgery
- ☐ Radiation therapy after surgery
- ☐ Radiation therapy BOTH before and after surgery
- ☐ Radiation therapy and NO surgery
- ☐ Immunotherapy
- ☐ Other
- ☐ No non-surgical treatments were offered
- ☐ I do not know/do not remember

7 [Q73]What type of surgical treatment did you initially receive for your DFSP or GCF? *

Please choose **only one** of the following:

- ○ Mohs' micrographic surgery
- ○ Wide local excision
- ○ Conservative excision
- ○ I have not yet had surgery. It is planned or will be planned
- ○ No surgery
- ○ I do not know or do not remember

© 2019 David MP et al. *JAMA Network Open.*

8 [Q74]After you were diagnosed with DFSP, what type of surgeon performed your first surgery intended to remove your DFSP or GCF? *

Only answer this question if the following conditions are met:
° ((**Q73.NAOK** == "1" or **Q73.NAOK** == "2" or **Q73.NAOK** == "3" or **Q73.NAOK** == "6"))

Please choose **only one** of the following:

- Dermatologist
- Surgeon specializing in Mohs' surgery
- General surgeon
- Plastic surgeon
- Surgeon specializing in sarcoma
- Other oncologist
- Orthopedic surgeon
- Other
- I do not know or do not remember

9 [Q75]If your doctor discussed your surgical margins with you before the pathology report returned, what size of margin did the surgeon remove surrounding your tumour? This is the size of margin the surgeon took before learning what the actual margin was from pathology. (Pick the value that is your SMALLEST margin) *

Only answer this question if the following conditions are met:
° ((**Q73.NAOK** == "1" or **Q73.NAOK** == "2" or **Q73.NAOK** == "3" or **Q73.NAOK** == "6"))

Please choose **only one** of the following:

- My surgeon knew they were unable to completely excise my tumour before the pathology report
- Less than 0.1 cm (1mm)
- Between 0.1cm- 0.5cm (1-5mm)
- Between 0.5-1 cm
- Between 1-2 cm
- Between 2-3 cm
- Between 3-4 cm
- Between 4-5 cm
- Over 5 cm
- I do not know or do not remember

© 2019 David MP et al. *JAMA Network Open.*

10 [Q76]Were your margins clear after your first surgery? (clear=negative for tumour) *

Only answer this question if the following conditions are met:
° ((**Q73.NAOK** == "1" or **Q73.NAOK** == "2" or **Q73.NAOK** == "3" or **Q73.NAOK** == "6"))

Please choose **only one** of the following:

- Yes (NO TUMOUR present at the margins)
- No (tumour WAS PRESENT at the margin)
- I do not know/I do not remember

11 [Q77]When your margins were clear (negative) after surgery, what was the distance of your nearest margin? (This information would be on your pathology report). *

Only answer this question if the following conditions are met:
° ((**Q73.NAOK** == "1" or **Q73.NAOK** == "2" or **Q73.NAOK** == "3" or **Q73.NAOK** == "6") and (**Q76.NAOK** == "1"))

Please choose **only one** of the following:

- Less than 0.5 cm
- Between 0.5-1 cm
- Between 1-2cm
- Between 2-3 cm
- Between 3-4 cm
- Between 4-5 cm
- Over 5 cm
- I do not know or do not remember
- Margins were not given on pathology report

12 [Q78]When you margins were NOT clear (positive) after your first surgery, what additional treatment were you offered? Select ALL that apply *

Only answer this question if the following conditions are met:
° ((**Q73.NAOK** == "1" or **Q73.NAOK** == "2" or **Q73.NAOK** == "3" or **Q73.NAOK** == "6") and (**Q76.NAOK** == "2"))

Please choose **all** that apply:

- Additional surgery
- Gleevec (imatinib)
- Radiation
- Immunotherapy
- Other treatment
- No additional treatment was offered

© 2019 David MP et al. *JAMA Network Open.*

13 [Q79]When your margins were NOT clear (positive) after your first surgery, what additional treatment did you RECEIVE (or plan to receive if you have not yet returned for more treatment)? *

Only answer this question if the following conditions are met:
° ((**Q73.NAOK** == "1" or **Q73.NAOK** == "2" or **Q73.NAOK** == "3" or **Q73.NAOK** == "6") and (**Q76.NAOK** == "2"))

Please choose **all** that apply:

- ☐ Additional surgery
- ☐ Gleevec (imatinib)
- ☐ Radiation
- ☐ Immunotherapy
- ☐ Other treatment
- ☐ No additional treatment was received

© 2019 David MP et al. *JAMA Network Open.*

Your DFSP or GCF or FS-DFSP treatment experience

14 [Q80]If you are currently cancer-free, how long have you been cancer-free? *

Please choose **only one** of the following:

- 3 months or less
- Between 3-6 months
- Between 6 months-1 year
- Between 1 year-2 years
- Between 2 years- 5 years
- Between 5-10 years
- Between 10-20 years
- Between 20 years-30 years
- More than 30 years
- Not applicable because I currently have tumour

15 [Q81]Did you ever require a skin graft? *

Please choose **only one** of the following:

- Yes
- No

16 [Q82]

Did you ever have a skin graft that failed? *

Only answer this question if the following conditions are met:
° ((**Q81.NAOK** == "1"))

Please choose **only one** of the following:

- Yes
- No

17 [Q83]Did you ever receive Gleevec (imatinib) for your DFSP or GCF? *

Please choose **only one** of the following:

- Yes
- No

© 2019 David MP et al. *JAMA Network Open.*

18 [Q84]What happened to the size of your tumour when you took Gleevec (imatinib)? *

Only answer this question if the following conditions are met:
° ((**Q83.NAOK** == "1"))

Please choose **only one** of the following:

- It decreased in size
- It stayed the same size
- It continued to grow (Gleevec/imatinib never made a difference)
- It responded for a while and then the Gleevec/imantinib stopped working
- It is too soon to tell
- Not applicable/I do not know

19 [Q85]I took Gleevec (imatinib)/I will take Gleevec (imatinib)... *

Only answer this question if the following conditions are met:
° ((**Q83.NAOK** == "1"))

Please choose **only one** of the following:

- Before surgery
- After surgery
- Both before and after surgery

20 [Q86]How long did you take/have you taken Gleevec (imatinib)? *

Only answer this question if the following conditions are met:
° ((**Q83.NAOK** == "1"))

Please choose **only one** of the following:

- 3 months or less
- Between 3-6 months
- Between 6 months-1 year
- Between 1 year-2 years
- Between 2 years- 5 years
- Between 5-10 years
- Between 10-20 years
- Over 20 years

© 2019 David MP et al. *JAMA Network Open.*

21 [Q87]Did you ever receive radiation treatment for your tumour? *

Please choose **only one** of the following:

- Yes
- No

22 [Q88]How did radiation treatment change the size of your tumour? *

Only answer this question if the following conditions are met:
° ((**Q87.NAOK** == "1"))

Please choose **only one** of the following:

- It decreased in size
- It stayed the same size
- It continued to grow
- It responded for a while and then the radiation stopped working
- It is too soon to tell
- Not applicable/I do not know

23 [Q89]I received radiation therapy/I will receive radiation therapy: *

Only answer this question if the following conditions are met:
° ((**Q87.NAOK** == "1"))

Please choose **only one** of the following:

- Before surgery
- After surgery
- Both before and after surgey

24 [Q91]Have you ever had recurrence? (Recurrence means the tumour grew back at the SAME location) *

Please choose **only one** of the following:

- Yes
- No

© 2019 David MP et al. *JAMA Network Open.*

25 [Q92]How many recurrences have you had? *

Only answer this question if the following conditions are met:
° ((**Q91.NAOK** == "1"))

Please choose **only one** of the following:

- ◯ 1
- ◯ 2
- ◯ 3
- ◯ 4
- ◯ 5
- ◯ More than 5

26 [Q91]If you have had pain at the site of your surgery after the site has healed, was this pain associated with a recurrence? (Recurrence means the tumour grew back at the same location) *

Please choose **only one** of the following:

- ◯ I have not had any pain at my surgical site
- ◯ I have had pain at my surgical site but it was not associated with a recurrence
- ◯ I have had pain at my surgical site and it was associated with a recurrence
- ◯ I do not know/I do not remember
- ◯ Not applicable

© 2019 David MP et al. *JAMA Network Open.*

27 [Q92]What symptoms did you have with any of your recurrences? *

Only answer this question if the following conditions are met:
° ((**Q91.NAOK** == "1"))

Please choose **all** that apply:

- ☐ I noticed no changes with my recurrence(s)
- ☐ Numbness/no sensation
- ☐ Tingling sensation
- ☐ Itchiness
- ☐ Dull painful/bruise-like feeling
- ☐ Sharp/stabbing pain
- ☐ Burning pain
- ☐ Coloration of normal skin
- ☐ Blue to purple discoloration
- ☐ Red discoloration
- ☐ Pink discoloration
- ☐ White/lighter than normal skin discoloration
- ☐ Ulceration/open sore
- ☐ The recurrence did NOT raise the surface of my skin
- ☐ Sensation of thickened area beneath the skin
- ☐ The recurrence DID raise the surface of my skin
- ☐ The recurrence felt like a lump/bump/knot
- ☐ The recurrence was a complicated lumpy/bumpy growth
- ☐ The recurrence was multiple bumps/nodules

28 [Q93]What treatment were you offered for your recurrence(s)? *

Only answer this question if the following conditions are met:
° ((**Q91.NAOK** == "1"))

Please choose **all** that apply:

- ☐ Additional surgery
- ☐ Gleevec (imatinib)
- ☐ Radiation
- ☐ No additional treatment was offered
- ☐ Not applicable

© 2019 David MP et al. *JAMA Network Open.*

29 [Q94]

What treatment did you receive for your recurrence(s)? *

Only answer this question if the following conditions are met:
° ((Q91.NAOK == "1"))

Please choose **all** that apply:

- ☐ Additional surgery
- ☐ Gleevec (imatinib)
- ☐ Radiation
- ☐ No additional treatment was received
- ☐ Not applicable

30 [Q90]Were you ever diagnosed with FS (fibrosarcomatous)-DFSP? *

Please choose **only one** of the following:

- ○ No
- ○ Yes, I had this diagnosis at my first biopsy or first excision
- ○ Yes, I had this diagnosis at a recurrence and before this I never had chemotherapy or radiation
- ○ Yes, I had this diagnosis at a recurrence and before this I had chemotherapy
- ○ Yes, I had this diagnosis at a recurrence and before this I had radiation
- ○ Yes, I had this diagnosis at a recurrence and before this I had chemotherapy and radiation
- ○ I do not know/do not remember

31 [Q95]Have you had any mestastases of DFSP or GCF or FS-DFSP either when first diagnosed or later? (Metastases means the tumour spread to another part of your body like brain, lungs, or other internal organs) *

Please choose **only one** of the following:

- ○ No
- ○ Yes, I had a metastasis of FS-DFSP
- ○ Yes, I had a metastasis of DFSP
- ○ Yes, I had a metastasis of GCF

© 2019 David MP et al. *JAMA Network Open.*

32 [Q96]Where were the metastases located (what part of the body)? *

Only answer this question if the following conditions are met:
° ((**Q95.NAOK** == "2" or **Q95.NAOK** == "3" or **Q95.NAOK** == "4"))

Please choose **all** that apply:

- ☐ Bones
- ☐ Muscle
- ☐ Lungs
- ☐ Heart
- ☐ Male reproductive system (prostate, testicles, penis)
- ☐ Female reproductive system (ovaries, uterus, vagina)
- ☐ Bladder
- ☐ Kidneys
- ☐ Liver
- ☐ Stomach
- ☐ Intestines
- ☐ Colon/Rectum
- ☐ Brain or neurological system (spine or nerves)
- ☐ Lymph node(s)
- ☐ Skin
- ☐ Spleen
- ☐ Other

33 [Q97]How were the metastases found? *

Only answer this question if the following conditions are met:
° ((**Q95.NAOK** == "2" or **Q95.NAOK** == "3" or **Q95.NAOK** == "4"))

Please choose **all** that apply:

- ☐ CT scan
- ☐ MRI
- ☐ PET scan
- ☐ X-ray
- ☐ Ultrasound
- ☐ Other imaging study
- ☐ Not applicable

© 2019 David MP et al. *JAMA Network Open.*

34 [Q98]After your surgery/surgeries, who provided follow-up exams to look for recurrence(s)? *

Only answer this question if the following conditions are met:
° ((**Q73.NAOK** == "1" or **Q73.NAOK** == "2" or **Q73.NAOK** == "3" or **Q73.NAOK** == "6"))

Please choose **only one** of the following:

- ◯ Primary care provider
- ◯ Dermatologist
- ◯ Surgeon
- ◯ Hematologist and/or oncologist
- ◯ I do not/I did not receive follow-up exams
- ◯ Not applicable
- ◯ I do not know/I do not remember

35 [Q99]How often do you receive/will you receive exams to look for recurrence(s)? *

Please choose **only one** of the following:

- ◯ Not applicable
- ◯ I do not know/I do not remember
- ◯ Follow-up exams will occur regularly for the rest of my life
- ◯ More frequent exams after surgery and then less frequent exams for the rest of my life
- ◯ More frequent exams after surgery, and then less frequent exams, and then "dismissed" from follow up
- ◯ I do not receive follow-up exams

36 [Q100]If you are answering on behalf of a deceased patient, did the patient die from complications of DFSP, GCF or FS-DFSP? *

Please choose **only one** of the following:

- ◯ No, the patient died from other causes
- ◯ Yes, the patient died from FS-DFSP
- ◯ Yes, the patient died from DFSP
- ◯ Yes, the patient died from GCF
- ◯ I am not answering on behalf of a deceased patient

© 2019 David MP et al. *JAMA Network Open.*

37 [Q101]

1. **If your tumour had a period of fast growth, were you taking any hormonal medication at that time? (For example, birth control pills, progesterone, estrogen, corticosteroids)?**

*

Please choose **only one** of the following:

- I have never noticed a period of fast growth of my tumour
- I have noticed a period of fast growth of my tumour, but I was not on any hormonal medications at that time
- Yes, testosterone
- Yes, birth control hormonal medication
- Yes tamoxifen
- Yes, another (not for birth control) progesterone or progesterone-related medication Yes,
- another (not tamoxifen or for birth control) estrogen or estrogen-related medication
- Yes, corticosteroids (dexamethasone, hydrocortisone, prednisone, for example)
- Yes, other not listed here
- I do not know or do not remember

38 [Q102]Had you STOPPED taking any hormonal medication within THREE MONTHS of when your tumour had a period of fast growth? (For example, birth control pills, progesterone, estrogen, corticosteroids)? *

Please choose **only one** of the following:

- I have never noticed a period of fast growth of my tumour
- No, I had not stopped taking hormonal medication
- Yes, testosterone
- Yes, birth control hormonal medication
- Yes tamoxifen
- Yes, another (not for birth control) progesterone or progesterone-related medication
- Yes, another (not tamoxifen or for birth control) estrogen or estrogen-related medication
- Yes, corticosteroids (dexamethasone, hydrocortisone, prednisone, for example)
- Yes, other not listed here
- I do not know or do not remember

© 2019 David MP et al. *JAMA Network Open.*

39 [Q102]When you were pregnant, did the growth rate of your tumour increase? *

Please choose **only one** of the following:

- I have never been pregnant
- Yes
- No

40 [Q103]In the 3 months after pregnancy, did you notice a period of rapid growth of the tumour? *

Only answer this question if the following conditions are met:
° ((**Q102.NAOK** == "2" or **Q102.NAOK** == "3"))

Please choose **only one** of the following:

- Yes
- No

41 [Q104]When you were breast-feeding, did you notice an increased rate of growth of the tumour? *

Please choose **only one** of the following:

- I have never breast-fed an infant/child
- Yes
- No

42 [Q105]Were you ever pregnant after being diagnosed? *

Please choose **only one** of the following:

- No
- Yes, and I did NOT experience a recurrence during pregnancy
- Yes, and I DID experience a recurrence during pregnancy

© 2019 David MP et al. *JAMA Network Open.*

Your medical and personal history

43 [Q106]Do you have any family members (brother, sister, parent, children) that also have received a DFSP/FS-DFSP or GCF diagnosis? *

Please choose **only one** of the following:

- ○ Yes
- ○ No

44 [Q107]Do you personally have a genetic cancer disorder? *

Please choose **only one** of the following:

- ○ No, or not that I know of
- ○ Yes, I have a BRCA mutation
- ○ Yes, I have FAP (Familial Adenomatous Polyposis)
- ○ Yes, I have Fanconi Anemia
- ○ Yes, I have Lynch syndrome/HNPCC (hereditary non-polyposis colon cancer syndrome)
- ○ Yes, I have Li-Fraumeni syndrome
- ○ Yes, I have a genetic cancer disorder not mentioned here

45 [Q108]Have you had any other cancer(s) other than DFSP? *

Please choose **all** that apply:

- ☐ No
- ☐ Yes, basal cell carcinoma
- ☐ Yes, melanoma
- ☐ Yes squamous cell carcinoma
- ☐ Yes, colon cancer
- ☐ Yes, uterine cancer
- ☐ Yes, brain cancer
- ☐ Yes, liver cancer
- ☐ Yes, pancreatic cancer
- ☐ Yes, a different sarcoma
- ☐ Yes, other cancer not mentioned here

© 2019 David MP et al. *JAMA Network Open.*

46 [Q109]Before you were diagnosed with DFSP or GCF, did you have chemotherapy for any other cancer? *

Only answer this question if the following conditions are met:
° ((**Q108_10.NAOK** == "Y") and (**Q108_11.NAOK** == "Y") and (**Q108_2.NAOK** == "Y") and (**Q108_3.NAOK** == "Y") and (**Q108_4.NAOK** == "Y") and (**Q108_5.NAOK** == "Y") and (**Q108_6.NAOK** == "Y") and (**Q108_7.NAOK** == "Y") and (**Q108_8.NAOK** == "Y") and (**Q108_9.NAOK** == "Y"))

Please choose **only one** of the following:

- ○ Yes
- ○ No

47 [Q110]Before you were diagnosed with DFSP or GCF, did you have any exposure to radiation (for example: CT scans, radiation therapy for another cancer, radiation exposure at work, pilot or airline attendant)? Select ALL that apply *

Please choose **all** that apply:

- ☐ Yes, CT scan(s)
- ☐ Yes, X-ray(s)
- ☐ Yes, Radiation exposure at work
- ☐ Yes, Radiation therapy
- ☐ Yes, Pilot or airline attendant
- ☐ Yes, Ultraviolet radiation
- ☐ Yes, Other type of radiation
- ☐ No

© 2019 David MP et al. *JAMA Network Open.*

48 [Q111] My DFSP or GCF healthcare has been received in: *

Please choose **only one** of the following:

- UK
- USA
- Puerto Rico
- Canada
- Mexico
- Australia
- China
- India
- Indonesia
- Brazil
- Pakistan
- Bangladesh
- Nigeria
- Russia
- Japan
- Germany
- Egypt
- France
- Italy
- Thailand
- Netherlands
- South Africa
- Ghana
- Other

49 [Q112]Did you have private medical insurance or did you use the National Health Service (NHS)? *

Only answer this question if the following conditions are met:
° ((**Q111.NAOK** == "1"))

Please choose **only one** of the following:

- Private medical insurance
- National Health Service (NHS)
- I have used both private medical insurance and National Health Service (NHS)

© 2019 David MP et al. *JAMA Network Open.*

50 [Q113]Did you switch from NHS to private medical insurance in order to see a provider sooner? *

Only answer this question if the following conditions are met:
° ((**Q111.NAOK** == "1") and (**Q112.NAOK** == "3"))

Please choose **only one** of the following:

- Yes
- No
- No, I switched for another reason
- Not applicable

51 [Q114]How long did it take you to receive a biopsy from the time you first went to a healthcare provider for your tumour? *

Only answer this question if the following conditions are met:
° ((**Q111.NAOK** == "1"))

Please choose **only one** of the following:

- 3 months or less
- 3-6 months
- 6 months to 1 year
- 1-2 years
- 2-5 years
- 5-10 years
- 10-20 years
- 20-30 years
- Over 30 years
- I do not know/I do not remember
- Not applicable

© 2019 David MP et al. *JAMA Network Open.*

52 [Q115]How long did it take you to receive an excision from the time you first went to a healthcare provider for your tumour? *

Only answer this question if the following conditions are met:
° ((**Q111.NAOK** == "1"))

Please choose **only one** of the following:

- 3 months or less
- 3-6 months
- 6 months-1 year
- 1-2 years
- 2-5 years
- 5-10 years
- 10-20 years
- 20-30 years
- Over 30 years
- I do not know/I do not remember
- Not applicable

53 [Q116]How many reconstructive surgeries have you had? *

Please choose **only one** of the following:

- 0
- 1
- 2
- 3
- 4-6
- 7-10
- 11-15
- 16-20
- 20 or more

© 2019 David MP et al. *JAMA Network Open.*

21.11.2015 – 00:00

Submit your survey.
Thank you for completing this survey.

© 2019 David MP et al. *JAMA Network Open.*
